# Designing a whole cell bioreporter to show antioxidant activities of agents that work by promotion of the KEAP1–NRF2 signaling pathway

**DOI:** 10.1038/s41598-019-39011-w

**Published:** 2019-03-01

**Authors:** Negar Mozahheb, Ehsan Arefian, Mohammad Ali Amoozegar

**Affiliations:** 1 Extremophiles Lab, Department of Microbiology, School of Biology and Center of Excellence in Phylogeny of Living Organisms, College of Science, University of Tehran, Tehran, 1417466191 Iran; 2 Department of Microbiology, School of Biology, College of Science, University of Tehran, Tehran, 1417466191 Iran

## Abstract

The major signaling pathway in human cells is related to the antioxidant defense system. The main component of this system is a transcription factor, Nuclear Factor Erythroid 2-Related Factor 2 (NRF2). It regulates this system in different cellular situations under stimulation by oxidative stress or antioxidants. Thus, detecting the stimulation of NRF2 via a screening strategy may enable us to discover stimulating agents of NRF2-related signaling pathway. With this in mind, we designed a whole cell bioreporter containing the NRF2 response elements that are inserted in a luciferase vector, immediately upstream of a luciferase gene whose promoter has been removed. This bioreporter is activated by stimulators such as 3H-1,2-dithiole-3-thione (D3T), butyl hydroxyanisole (BHA) and ascorbic acid reacting as antioxidant agents. It was observed that the regulatory region of the NRF2 gene, which is identified by NRF2 protein, is located inside its coding region. This designed bioreporter can detect the presence of antioxidant agents. It also exhibits a significant linear correlation over different doses of these agents ranging from 0.8 to 80 μM for ascorbic acid, 0.1 to 100 μM for D3T, and 0.1 to 100 μM for BHA. This detection system is proven to be more sensitive than Real-time PCR, suggesting it to be a highly sensitive system among the available methods.

## Introduction

One of the most studied stresses that threatens the stability of a diverse range of cells is oxidative stress, which causes numerous damages in cells and leads to several organs’ dysfunctionalities and disease such as cancer, neurodegenerative disease, retinopathy, dermatological disease, etc. In general, upon being exposed to any given stress, cells try to maintain their cellular homeostasis to keep the basic internal permanent condition, and likewise, upon oxidative stress, cells move toward maintaining their redox homeostasis^1–3^. To do so, cells benefit from a major signaling cascade that provides antioxidant and detoxification defense to almost all human cells. The antioxidant defense system is a major protective mechanism that reduces the stress-induced damaging effects via neutralizing the oxidants and electrophiles using antioxidants. It benefits from an important component, NRF2 (Nuclear Factor Erythroid 2-Related Factor 2), which is a transcription factor and a member of the cap ‘n’ collar (CNC) subfamily of basic region leucine zipper (bZip) transcription factors. The key role of NRF2 in controlling cellular defense against environmental oxidant agents has been revealed by studies in which NRF2-knocked-out mice have been shown to exhibit sensitivity to hyperoxia-induced injury, as well as increased susceptibility to toxic xenobiotic, including carcinogens^4,5^.

Oxidative stress could be imposed by endogenous conditions and several exogenous factors, which in part lead to the promotion of the mentioned regulations and gene activations. For instance, UV-irradiation, drugs, and chemicals such as chemotherapeutic drugs can create free radicals both in external cellular microenvironment as well as cells’ internal spaces^6,7^. During the first stage of an oxidative stress, NRF2 is activated via the disassociation of NRF2 from its repressor protein in the cytoplasm, KEAP1, which contains cysteine residues. In detail, KEAP1 reacts with oxidative and electrophilic radicals leading to conformational changes and the release of NRF2. Subsequently, the translocation of NRF2 to the nucleus takes place and it binds to Antioxidant Response Element (ARE) resulting in the transcription of defensive genes^8,9^. The activation of the transcription involves NRF2 recognizing its own promoter and establishing an effective interaction with it and the newly formed and accumulated NRF2 in the nucleus binds to promoters of other specific genes. Such genes encode detoxifying enzymes/proteins including Glutathione-S-Transferases(GSTs), Superoxide Dismutase(SOD), Catalase, NAD(P)H: Quinoneoxidoreductase-1(NQO1) as well as stress response proteins such as heme oxygenase-1 (hmox1) and -2 (hmox2), metallothioneins and heat shock proteins. These proteins provide cellular protection against various oxidants or pro-oxidant attack^10,11^.

In addition to the activation of NRF2 by exogenous and endogenous stresses, almost all antioxidant chemicals, such as carotenoids, can interestingly activate NRF2 protein as a transcription factor as well^12,13^. Antioxidants perform as the accelerator of this protective system through two major mechanisms: first, they have specific functional groups that are capable of disrupting the NRF2-KEAP1 complex leading to the release of the latter part form the former. This happens via changing the conformation of the KEAP1 and disrupting the ubiquitination of the NRF2 which result in successful transcription of the antioxidant defense gene^14^. Second, they can also act as free radical scavengers which results in the neutralization of the oxidants^9^. That means that they can neutralize the free radicals such as reactive oxygen and nitrogen species via reducing them to stable compounds and break the molecular chain oxidation reactions, both in cells and extra-cellular environments^15^.

NRF2 can get activated by both oxidants and antioxidants which is unique. It is worth mentioning that most of NRF2-pathway inducers are KEAP-1 inhibitors^16,17^. There are almost ten chemical groups that interact with the thiol groups of cysteines in KEAP-1. One of such groups is polyphenolic compounds that have been reported to exhibit antioxidative activates. BHA is a known member of this category which protect cells from oxidative stress via either scavenging free radicals and/or induction of the KEAP1-NRF2 pathway^16,18,19^. Another chemical group is Dithiolethiones, and a natural example of this category is D3T with similar function to BHA^20^. There are inhibitors for NRF2 as well, such as ascorbic acid (Vitamin C). They reduce peroxide level and suppresses the formation of NRF2/DNA complex at the ARE regions of antioxidative and cytoprotective genes, especially the glutamate-cysteine ligase (GCL)^21,22^.

There are some reports on the development of antioxidant detection methods. Some of these methods such as DPPH^23^ and ABTS^24^ assays are *in-vitro* systems based on evaluating the neuteralization of free radicals. Other methods involve evaluating the antioxidant activities using markers for oxidation-induced tissue damage, such as lipid peroxides^25,26^. On the other hand, there are several *in-vivo* antioxidant detection systems. The majority of such methods are related the effects of antioxidants on the activation of the KEAP1-NRF2 pathway, while other such approaches focus on pathways that are not related to this pathway directly. An example of the later includes measuring the antioxidant-induced suppression of protein carbonylation^27^.

There are some biases in these methods. For example, in *in-vitro* methods, the antioxidant activity in neutralizing the free radical and electrophiles is interpreted as a direct antioxdant effect, assuming that the agents do not activate the cell defense system^28^. Such methods are suitable only for evaluating agents that don’t have any effects on cells’ antioxidant defense system^29^. Some other methods, such as KEAP1-NRF2 independent *in-vivo* method, the antioxidant activity is evaluated based on the agent’s ability to induce the production of antioxidant agent such as glutathione, while their direct antioxidant activity is assumed to be neglected^30^. Methods based on the activation of KEAP1-NRF2 pathway suffer from the same issue. It is worth mentioning that if the mentioned assumptions do not stand, obtained results might be bias.

**Figure 1:**
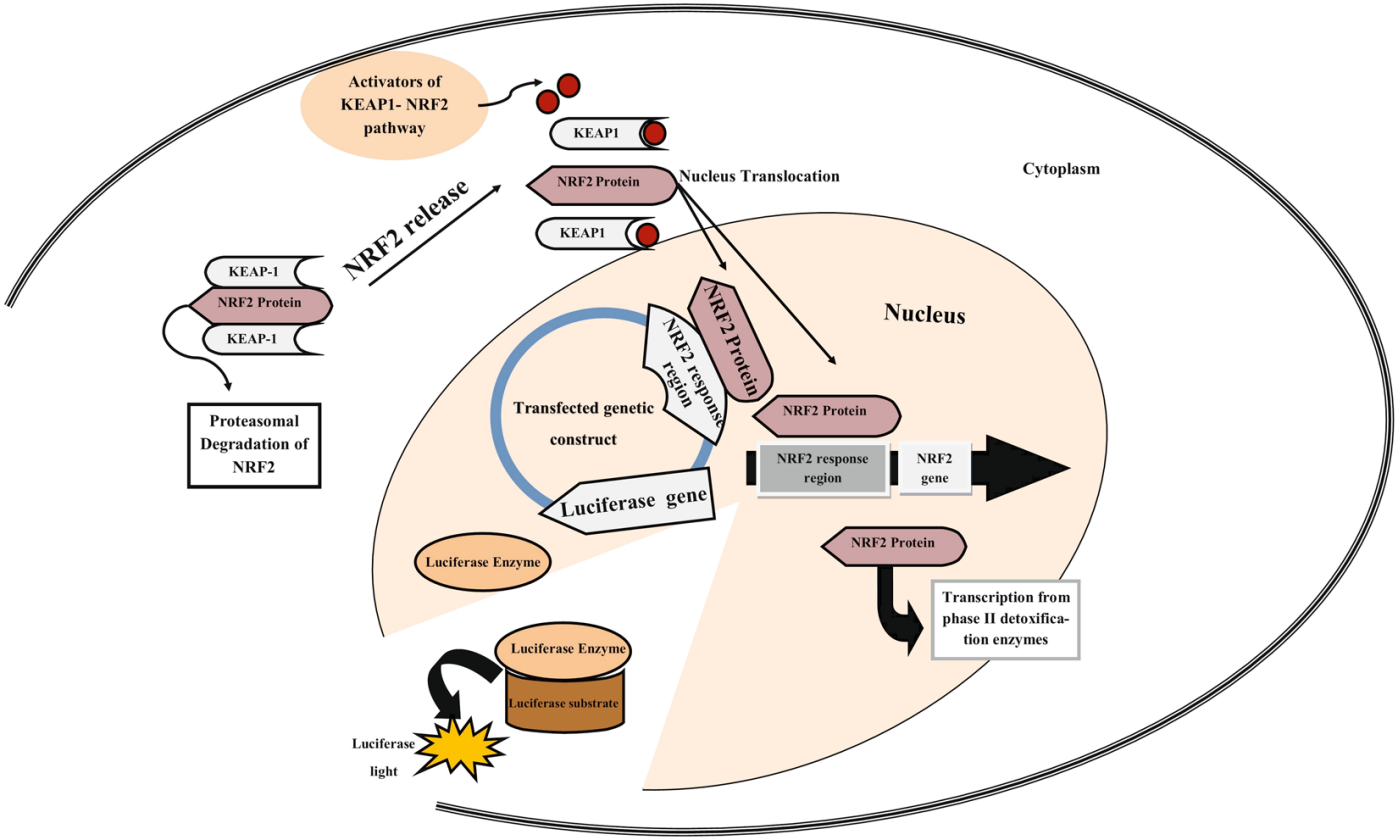
Schematic of our bioreporter. During NRF2-KEAP1 pathway activation, NRF2 is disassociated from its repressor protein in the cytoplasm, KEAP1, followed by its translocation to the nucleus, binding to its ARE at the upstream of the NRF2 gene and also that of transfected vector’s luciferase.

Given that NRF2 is the most significant protein in a cell’s antioxidant defense system, and the activation of its promoter is one of the first steps in NRF2 signaling pathway, measuring the activation of NRF2 promoter may provide a smart clue for evaluating the oxidant or antioxidant activities of drugs (i.e. their capability to promote the KEAP1-NRF2 signaling pathway). Thus, we focused our attention to benefit from this idea and design a molecular structure as a biosensor to detect oxidant or antioxidant activities of components (Fig. 1). We need to emphasize that the designed bioreporter responses to the release of NRF2 regardless from the release pathway. This means that the bioreporter can as well report on antioxidants that do not directly interact with KEAP1-NRF2, but rather activate pathways that result in the release of NRF2. We moved on to study the dose-dependent response of this biosensor using some well-known antioxidant agents such as BHA, D3T and ascorbic acid. We suggest that this detection system can be used for other known or unknown components to estimate their antioxidant activity. There are a lot of drugs, natural products, and cosmetics that have antioxidant effect^31,32^. This bioreporter can be highly useful to evaluate the role of agents in the activation of this pathway, making it potentially valuable in pharmaceutical and cosmetics industry.

## Results

### Effects of antioxidants in different doses on expressions of NRF2

**Figure 2:**
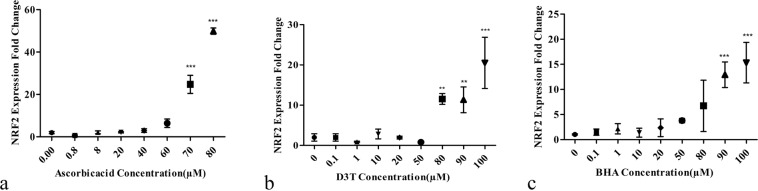
Effects of different concentrations of antioxidants on NRF2 expression. The meaningful overexpression of the NRF2 at concentrations of about 80, 90 and 100 µM of ascorbic acid (**a**), 80, 90 and 100 µM of D3T (**b**), and 90 and 100 µM of BHA (**c**). Values reported are the average ± SD of three independent experiments. ***P-value < 0.001, **P-value < 0.01 using One-way ANOVA data analyzing.

To evaluate the effect of concentrations of real antioxidants such as ascorbic acid, BHA and D3T on the expression of the NRF2_,_ in primary skin fibroblast cells, the cells were treated with these agents in different concentrations. It was observed that at most effective concentrations of antioxidants in general, the NRF2 expression was increased compared to non-treated cells. Ascorbic acid at concentrations of about 70 and 80 μM can significantly increase the expression of the NRF2_,_ but at lower concentrations, no change in the expression of the NRF2 in treated cells compared to non-treated ones was observed (Fig. 2). Similar results were also observed for cells treated with different concentrations of BHA and D3T. It was observed that in the cells that were treated with 80, 90 and 100 µM of D3T and BHA, the expression of NRF2 compared to that in non-treated cells significantly increased. (Fig. 2).

### Western blotting analysis confirmed the Real-time PCR results

**Figure 3:**
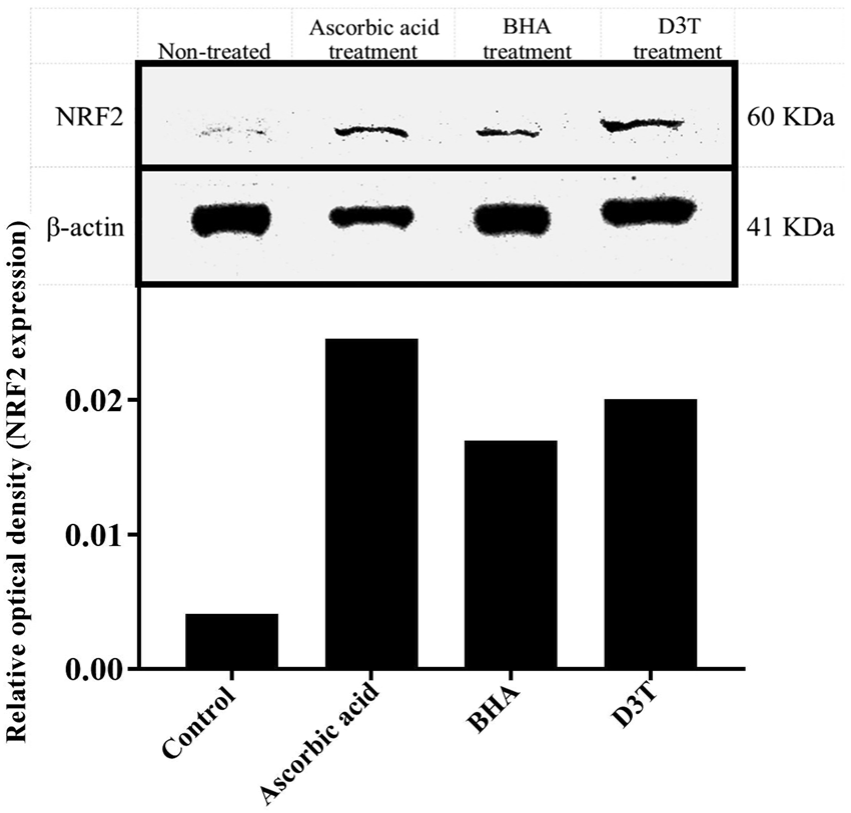
Western blot analysis shows an increase in NRF2 protein level in cells. The cells were treated with ascorbic acid (80 µM), BHA (100 µM), and D3T (100 µM) and after 12 hours the NRF2 protein level was compared to non-treated control. The graphs are related to optical density of NRF2 bands that are normalized by the internal control (β-actin). Full-length blots with molecular marker are presented in Supplementary Fig. S1.

To find out whether these antioxidants can impact the NRF2 protein level at their maximum effective concentrations, western blotting analysis was performed. The cell lysates were extracted after 12 hours from treated cells (corresponding to the time for RNA extraction for quantitative-PCR). Western blotting results were consistent with those from Real-time PCR. In other words, ascorbic acid, BHA and D3T at their maximum effective concentrations (80, 100 and 100) after 12 hours of incubation, not only increase NRF2’s mRNA level in primary skin fibroblast cells, but they also raise the NRF2 protein level (Fig. 3).

### The chosen genetic construct respond to the presence of antioxidants

**Figure 4:**
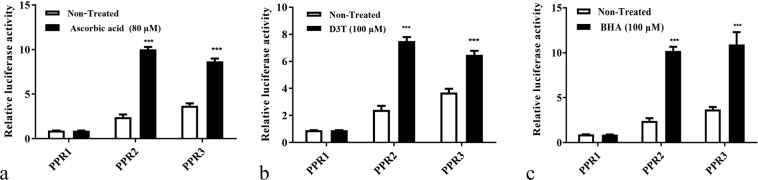
Comparative graph showing effects of maximum concentrations of antioxidants, (**a**) ascorbic acid (80 µM), (**b**) D3T (100 µM), and (**c**) BHA (100 µM) on three transfected HEK 293 cells with different genetic constructs, using Eq. 1. Reported values are the average ± SD of three independent experiments and they indicate better induction of PPR2 when affected by ascorbic acid. ***P-value < 0.001 by Two- way ANOVA data analyzing.

While looking to find regions containing response elements, it was observed that in both non-treated and treated (with ascorbic acid, D3T, and BHA) control group cells, no luciferase expression was observed. However, some luciferase activity was seen in non-treated, transfected with PPR-2 and PPR-3 cells, the amount of which was expectedly lower than that of the treated ones. As seen, at the concentration of ascorbic acid with the maximum-effectiveness (based on Real-time PCR and western blotting results), there was no significant difference in relative luciferase activity between treated and non-treated cells transfected with PPR-1. However, when PPR-2 and PPR-3 were transfected, cells that were treated with maximum effective concentration of ascorbic acid show luciferase activity with a significant difference compared to non-treated cells. Almost identical results were observed for BHA and D3T (Fig. 4). In other words, cells that were transfected with PPR-1 didn’t respond to these three putative antioxidant agents. However, cells transfected with PPR-2 and PPR-3 showed a significant luciferase activity response upon being treated with the mentioned antioxidants. Furthermore, comparing the effects of the antioxidants on the transfected cell groups using equation 1, it seemed that PPR-2 is more sensitive, and also upon being treated, could be induced more due to its greater change in luciferase activity compared to its non-treated cells (Table 1). Thus, it was chosen for the next step, i.e. evaluation of the dose-dependent activity of the bioreporter against different doses of antioxidants.

**Table 1:** A comparison of the normalized relative luciferase activities in different groups of transfected cells compared to their respective controls, using Eq. 2.

Groups	Treatments		
	Ascorbic acid (80 µM)	D3T (100 µM)	BHA (100 µM)
PPR1	0.988	0.991	1.003
PPR2	4.162	4.243	3.115
PPR3	1.443	1.078	1.755

Results show more sensitivity for PPR-2 construct.

### Dose-dependent activity of the bioreporter

**Figure 5:**
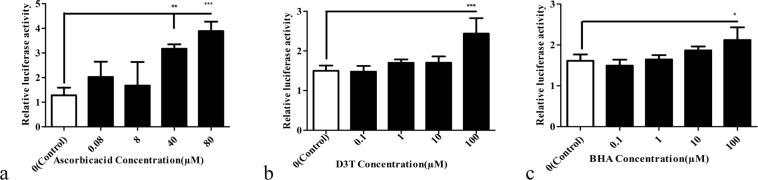
Effects of different concentrations of antioxidants on the activity of NRF2 response region, using Eq. 1. HEK 293 cells were transfected with PPR2 and then treated with (**a**) 0.8, 8, 40 and 80 μM of ascorbic acid, (**b**) 0.1, 1, 10 and 100 μM of D3T, and (**c**); 0.1, 1, 10 and 100 μM of BHA. Reported values are the average ± SD of the three independent experiments. ***P-value < 0.001, **P-value < 0.01, and *P-value < 0.5 by One-way ANOVA data analyzing.

It would ideal for our bioreporter to respond to antioxidants in a dose-dependent manner. Thus, the responses of the bioreporter to different doses of the antioxidants were evaluated. PPR-2 was chosen for this purpose as it exhibited the highest sensitivity. The vector containing this fragment was transfected to cells, the cells were treated with different concentrations of the three antioxidants mentioned above. According to the results (Fig. 5), out of the four concentrations that were studied for ascorbic acid, only two of them (80 and 40 µM) had a significant effect on cells to activate their luciferase promoter.

D3T and BHA also induced luciferase activity at the concentration of 100 µM (Fig. 5).

**Figure 6:**
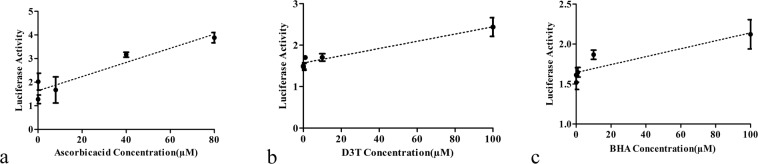
The luciferase activity of bioreporter against different concentrations of antioxidants (**a**) ascorbic acid (0.8, 8, 40 and 80 μM), (**b**) D3T (0.1, 1, 10 and 100 μM), and (**c**) BHA (0.1, 1, 10 and 100 μM). The results show a linear relationship between luciferase activity and doses of the antioxidants.

The average effectivities of the antioxidants on the bioreporter were also calculated and a linear regression model for bioreporter effectiveness at different concentrations of the antioxidants was plotted. The results reveal that although according to one-way ANOVA, only two concentrations of ascorbic acid and one concentrations of D3T and BHA showed significant difference in activity compared to their respective controls, but the linear regression analysis of the results represents a meaningful linear relationships between the antioxidants concentrations and the luciferase activity (Fig. 6, Table 2).

**Table 2:** Regression stimation results for Fig. 6 graph confirm the significant linearity between luciferase activity of bioreporter and doses of antioxidants.

*P*-Value	r^2^	Coefficient of independent variable(Dose)	Treatments
<0.0001	0.739	0.03 ± 0.005	Ascorbic acid (80 µM)
<0.0001	0.765	0.009 ± 0.001	D3T (100 µM)
0.0018	0.540	0.005 ± 0.001	BHA (100 µM)

## Discussion

We have designed a whole cell bioreporter that can sense activities of antioxidant using their influence on the most important transcription factors, NRF2. In fact, in the steady state of cells, KEAP1 proteins not only inhibit the NRF2 nuclear translocation, but they also lead to its ubiquitination and degradation, whereas the increase in cytosolic free NRF2 may result in its nuclear translocation and NRF2/DNA complex formation. It is a known fact that dithiolethiones including D3T and also phenolic antioxidants such as BHA can release NRF2 protein from its inhibitors, resulting in the expression upregulation of NRF2’s and other antioxidants’ genes^16,33^. In this study, ascorbic acid, D3T, and BHA were used to ensure the proper operation of the designed bioreporter for the detection of the NRF2 signaling pathway induction owing to their ability to affect the mRNA and protein level of NRF2.

In previous studies, there has been a focus on utilizing the Antioxidant-Response Elements (ARE) in the upstream region of the nucleotide sequence of NRF2 and phase 2 antioxidant enzymes to detect the activation of NRF2 signaling pathway. In other studies, the repeated nucleotide sequences containing ARE that can interact with the NRF2 transcription factor were used. The NRF2 signaling pathway can be activated by different antioxidant agents and the detection of such pathway activation was interpreted as an indication for the presence of antioxidants^33,34^. In our study, ENCODE database was used, which tries to search within the human genome to identify all functional elements. The identified sites would be recognized by a given transcription factor (such as NRF2) based on biochemical moedifications that have taken place in those areas. Interestingly, ENCODE is granted the access to UCSC consortium, thus their data are in accordance with each other, and ENCODE outputs could be transferred onto UCSC for further analysis^35^.

In ENCODE project database, two positions are suggested where histone modification takes place and is identified by NRF2 as a transcription factors: the first one contains 666 nucleotides, and has been located near the transcription start site (TSS) of NRF2 gene, and is also part of the first exon of NRF2 gene (+75/+741). The second region is located a little upstream from the former (−728/+152). To find out the effective part of these regions with promoter activity using bioinformatic analysis, this region was divided into three separate sequences, namely PPR1 (−728/+152), PPR2 (+75/+460), and PPR3 (+75/+741). These three sequences were inserted on the upstream of the luciferase gene. Such construct is then expected to exhibit luciferase activity upon activation of by NRF2. Since NRF2 gets translocated to the nucleus when exposed to antioxidants, luciferase activity is interpreted as signal to detect antioxidants.

Our first empirical step in our study was to test the Hu-02 cells using Real-time PCR and western blotting to check if they express NRF2, both in mRNA and protein levels, when exposed to antioxidants (ascorbic acid, D3T, BHA). The Real-time PCR results of this study showed that different concentrations of ascorbic acid (70 and 80 μM), D3T (80, 90 and 100 μM) and BHA (90 and 100 μM) increased the level of NRF2 mRNA only after 12 hours of incubation (and probably less). However, at lower concentrations of these antioxidants, there are no significant differences between NRF2 mRNA concentrations in both treated and non-treated cell groups. In other words, at lower concentrations of the antioxidants, the increase in the expression of the NRF2 is too low to be detected after 12 hours with Real-time PCR. On the other hand, western blotting analysis is in agreement with the Real-time PCR results at maximum effective concentrations of the antioxidants. These results proved that the Hu-02 cells were the excelent candidate for our study as they respond to the presence of antioxidants. In several studies on cells’ antioxidants defense systems, the experiments have been done on cells that are most exposed to oxidative stress, such as retinal cells^36^, lens epithelial cells^3^, or the cells that have the role of regeneration or protection in specific tissues such as fibroblasts^37^. In this study, we used skin fibroblast cell. The skin acts as a physiological barrier to protect the organism against environmental threats, one of which is oxidative stress, and thus, NRF2 expression in these cells is highly regulated. Thus, the antioxidants effects of candidate compounds were evaluated on the skin primary fibroblast cell. The antioxidant activities of various agent have already been evaluated on human dermal fibroblasts as well^8,9^. Also, we use primary cells as they have no genetically and morphologically modifications, especially at their primary passages compared to their immortal cell lines. We also use their DNA to separate regulatory regions of the NRF2 gene for designing the bioreporter. As mentioned, luciferase activity is expected to increase in the presence of antioxidants. To transfect the cells with designed constructs, immortal lines are better choice than primary cells because the transfection is easier and there is potential to create more stable reporter. HepG2 (human hepatoblastoma)^34^ and MCF7 (human breast carcinoma)^38^ are examples of used cell lines. We use HEK 293 cell line for luciferase assay because of the following advantages of these cells; Being easily grown in serum-free suspension culture, having quick reproduction and maintenance, being amenable to transfection with lipofectamine, being highly efficient at transfection and protein production, and being suitable for transient expression of protein (luciferase enzyme in our experiment).

Luciferase activity results showed that among these three candidate regions, PPR-2 and PPR-3 could indeed respond to the presence of antioxidants. Surprisingly enough, PPR-2 and PPR-3 have no ARE with putative sequence (5′-TGACnnnGCA-3′) which were worked on and discussed in several related articles^9,20,39,40^. PPR-1 on the other hand, has some ARE-like sequences and didn’t show any promoter activity upon exposure to antioxidants. However, in a previous study murine NRF2 promoter (−1065/−35) was utilized which contained ARE sequences^41^. This contrast may be related to the NRF2 gene regulation under the control of the probable alternative promoter regions in the first exon, or some alternative modulation such as DNA methylation in the coding region of NRF2 gene^42,43^. Moreover, with a slight difference, PPR-2 with its shorter sequence showed more susceptibility against antioxidants. Thus, one can conclude that the most effective NRF2 response regions are located in this part.

As NRF2 gene has ARE that reacts with NRF2 protein, it is expected that antioxidants, which are direct inducers of NRF2, must have the ability to induce the NRF2 promoter and increase its transcription. Conversely, antioxidant agents which act as the inhibitors of NRF2 activation may lead NRF2 to connect with KEAP1 proteins and get inhibited. D3T and BHA are the inducers of NRF2 through the KEAP1-NRF2 signaling pathway, whose antioxidant role has been discussed in several papers^6,9,20^. In some studies, ascorbic acid at specific concentrations has been considered as an inhibitor of NRF2 owing to its free radical scavenging properties and the ability to maintain the cellular redox status without stimulating NRF2. Reduction of cytosolic free radicals leads to the release of NRF2 from DNA followed by joining its cytoplasmic inhibitors^16^. However, what was observed was in contrast to the previous report: An increase in NRF2 mRNA and protein levels at the maximum effective concentration of ascorbic acid, and also an enhancement in the NRF2 promoter activity. This may be due to the ascorbic acid oxidation and its conversion into ascorbate monoanion (Asc°-). As a matter of fact, in the presence of transition metal cations, ascorbate ion can reduce trivalent iron ion to its divalent form, while getting oxidized into dehydroascorbate. Within the cells, dehydroascorbate is rapidly reduced back to ascorbate by GSH- and NAD(P)H-dependent reactions^19,44^. To maintain the required concentration of the GSH and NAD(P)H in the cells, they are reduced back into their original form through the activity of GSTs and NQO1 enzymes. Thus, as these enzymes are activated by the NRF2 transcription factor, cell treatment with ascorbic acid may increase NRF2 nuclear translocation, leading to an influence on the promoters of phase II detoxification genes, one of which being NRF2, which in turn results in its overexpression^45,46^.

Another notable point in the results of this study was a meaningful linear correlation between several doses of antioxidants and the luciferase activity of transfected cells. The concentration ranges of our study were from 0.8 to 80 µM for ascorbic acid, from 0.1 to 100 µM for BHA, and from 0.01 to 100 µM for D3T. However, in several other studies, different concentrations for these agents are used^27,46^.

Even though a wide range of concentration was used to find the linear response of the bioreporter, only the maximum non-toxic concentrations of BHA and D3T, and the maximum, and half maximum concentrations of ascorbic acid promoted NRF2 response elements significant to respond compared to the non-treated control, probably because the NRF2 protein retains its activity in non-treated cells without antioxidants induction^20^. While the effects of these antioxidants on activation of the NRF2 response elements have a linear relationship over a wide concentration range, Real-time PCR exhibits sensitivity only at high concentrations of antioxidants. This may have to do with the different mechanisms an antioxidant can have an effect on NRF2 as the first element in antioxidant defense pathway. Increasing the NRF2 protein stability in the cytoplasm by inhibition of its proteasomal degradation is one of the other ways antioxidants function^14^.

Another advantage of our probe is revealed when the mechanism of the process is analyzed more carefully. When an antioxidant enters a cell, NRF2 is released from its complex with KEAP-1, and gets translocated to the nucleus and targets our construct as well as the NRF2 promoter in the cell’s gene. The activation of our bioreporter results in the production of luciferase which generates an instant emission signal. Using Real-time PCR, on the other hand, involves amplification of the transcripted mRNA molecules which makes the whole process more tedious, challenging, and time-consuming.

We can infer from these results that the bioreporter we designed, which doesn’t contain any ARE sequence, can sense not only the presence of antioxidants but can also differentiate between various concentrations of these agents. Previous studies reported similar results even though their reporters benefited from ARE sequences in similar genetic constructs^34^.

Finally, we designed a whole cell luciferase base bioreporter based on the NRF2 response regions. Our bioreporter can get activated by NRF2 and is induced by antioxidants through the KEAP1-NRF2 signaling pathway. This bioreporter works based on the luminescence activity that can be induced by the antioxidants. Also, it can show different responses with a linear relationship to the concentration of the effective agents. Actually, our bioreporter can act not only in a qualitative manner but also in a quantitative manner. This is highly valuable as this feature can be used in several natural products and pharmaceutical industries to find antioxidant activities of agents through the activation of the KEAP1-NRF2 signaling pathway with no need for additional tests.

## Materials and Methods

### Chemicals

RNA Extraction and cDNA synthesis kit (Fermentas™), High rox, AMPLICON™ Real-time PCR master mix, PCR reagent and restriction enzymes, penicillin/streptomycin solution, Pierce™, NRF2 antibody(sc-365949), β-actin antibody (ab8227), anti-mouse IgG (ab6728), *PageRuler* prestained protein ladder 10 to 180 kDa (Fermentas™), luciferase kit, ECL kit (Millipore), pRL and pGL.4.14 (Promega™). DMEM cell culture medium and fetal bovine serum (FBS). lipofectamine 2000 (Invitrogen™), BHA, ascorbic acid, D3T.

### Cells

Human primary fibroblast skin cell (Hu-02) and HEK 293 were purchased from IBRC (Iranian Biological Resource Center).

### Cell culture

Cells were cultured in DMEM supplemented with 10% (v/v) FBS and 1% (v/v) penicillin/streptomycin solution (Invitrogen-Gibco) at 37 °C in a humidified atmosphere of 5% CO2. For Hu-02 cell of passage 3 to 5 were used.

### Quantitative PCR

The cells were seeded in a 12-well plate. When the cells reached enough confluency (lower than 2 × 10^5^ cell per well), they were treated with different concentrations of ascorbic acid (0.8, 8, 20, 40, 60, 70, 80 μM), BHA (0.1, 1, 10, 20, 50, 80, 90, 100 μM) and D3T (0.1, 1, 10, 20, 50, 80, 90, 100 μM). After 12 hours, RNA was extracted from different groups of treated cells, corresponding cDNA synthesis was done and quantitative PCR performed on 7500 real-time PCR System (Applied Biosystems), using cells’ cDNA, exon-exon junction primers of the NRF2 and ß2M as the housekeeping genes (Supplementary Table S1 online). The relative quantities were determined using the ΔΔC_T_ method^47^.

### Western blotting

The cells were seeded in a 12-well plate. After the cells reached enough confluency (lower than 2 × 10^5^ cell per well), they were treated with 80 μM of ascorbic acid, 100 μM of BHA and 100 μM of D3T. Groups of two wells were used and treated similarly. The cell extracts were prepared with RIPA lysis buffer. After one hour of shaking at 4 °C and centrifugation (14000 RPM for 15 min), the protein concentration of each group was measured by BCA assay and equivalent amounts of protein were used for consequence steps. The supernatant cell extract (30 µl) was mixed with sodium dodecyl sulfate (SDS) sample buffer (50 mM Tris–HCl [pH 6.8], 10% glycerol, 2% SDS, 0.1% bromophenol blue, and 5% β-mercaptoethanol) and separated by 8–10% SDS-polyacrylamide gel electrophoresis followed by transferring to a PVDF membrane (0.45 µm; Roche). After incubation with blocking solution (5% skimmed milk in Tris-buffered saline with 0.1% Tween-20 [TBS-T]) for 1 hours at room temperature (RT), the protein bands were probed by mixing the membrane with NRF2 antibody (1:100), β-actin antibody (1:100) and incubating overnight at 4 °C. HRP-conjugated rabbit anti-mouse IgG antibody (1:100) was used as a secondary antibody. Immune complexes became visible using the ECL kit. β-actin was used as an internal standard to standardize the protein amounts loaded to each lane. The density of protein bands was quantified by Tatallab software.

### Bioinformatics studies

The three genetic constructs are chosen on the basis of NRF2 promoter activation. For this purpose, the NRF2 promoter regions were determined. Initially, three potential regions with the capability of promoting NRF2 gene were found according to the literature and bioinformatics tools. The two main bioinformatics tools for gene sequences and transcription factors employed in our study are the UCSC database (https://genome.ucsc.edu/cgi-bin/hgGateway) and ENCODE (https://www.encodeproject.org/), respectively.

**Figure 7:**
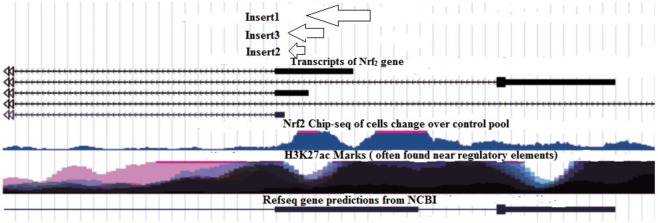
Image of UCSC output. Insert 1, Insert 2 and Insert 3 are probable promoter regions including those that were cloned.

NRF2 protein was found to be a transcription factor in ChIP-seq ENCODE date. Since NRF2 has been reported to promote its own gene, it was then tried to pin-point the region NRF2 binds to promote its own gene by transferring the ENCODE data to UCSC. This resulted in finding regions that are recognized by NRF2 as promoter and at the same time code for the NFR2 protein (Fig. 7, Inserts 1, 2, and 3). The three regions (Fig. 7) were amplified by PCR from the genomic content of Hu-02 cells (see Supplementary, Table S1), and cloned separately into a suitable luciferase reporter vector using the HindІІІ-XhoІ restriction enzyme in a head-to-tail orientation at the upstream of the luciferase gene transcriptional unit.

### Genetic constructions

**Figure 8:**
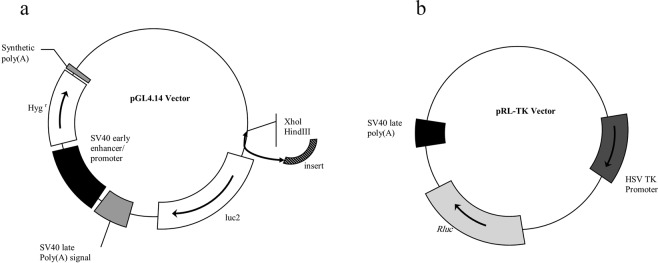
Vector maps. (**a**) pGL4.14 vector, (**b**) pRL-TK vector.

The luciferase vector that was selected for this purpose was pGL4.14 (Fig. 8). pRL-TK vector was used as an external control (Fig. 8) containing a different luciferase gene from that of pGL4.14. The pRL-TK vector comes with its own promoter to correct for the data alterations that may have been caused during the transfection procedure.

### DNA transfection and cell treatments

HEK 293 cells were seeded in a 96-well plate at 7 × 10 ^3^ to 8 × 10^3^ cells/well. The cells were incubated overnight in an antibiotic-free DMEM media, followed by performing transfection at 60% confluency as follows: Four groups of cells were defined as **PPR-1**, **PPR-2**, **PPR-3**, and a **control group**, and transfected with pGL4.14/insert 1 and the pRL-TK control, pGL4.14/insert 2 and pRL-TK control, pGL4.14/insert 3 + pRL-TK control, and pGL4.14 with no insertion + pRL-TK control, respectively (inserts are shown in Fig. 7). In all groups, cells were transfected with 1 µg of pGL4.14 and 0.1 µg of pRL-TK plasmids in each well, using lipofectamine 2000. After 18 hours, cells were treated with the most effective concentrations of ascorbic acid (80 μM), BHA (100 μM) and D3T (100 μM) to evaluate which constructs were induced when exposed to antioxidants.

To find the detection limit of this bioreporter system, cells that were transfected with the most sensitive genetic construct were treated by 4 different concentrations of ascorbic acid (0.8, 8, 40 and 80 μM), BHA (0.1, 1, 10 and 100 μM), D3T (0.1, 1, 10 and 100 μM), and no treatment for the control. After 12 hours of incubation, cells were lysed. *Firefly* and *Renilla* luciferase activities were measured using dual-luciferase kit Promega™. Eq. 1 was used to obtain the relative luciferase activity.

1 $$Relative\,luciferase\,activity=\frac{Average\,of(Firefly)}{Average\,of(Renilla)}$$

Eq. 2 was used to determine the normalized relative luciferase activities between test groups.

2 $$\begin{array}{c}Normalized\,\\ luciferase\,activities\end{array}=\frac{{\{AverageofFireflyactivity/AverageofRenillaactivity\}}_{treated}}{{\{AverageofFireflyactivity/AverageofRenillaactivity\}}_{non-treated}}$$

### Statistics

All experiments were repeated three times, and the results were reported as mean ± standard deviation. Comparisons between samples were carried out using Two-way ANOVA and One-way ANOVA with a statistical significance at *P* < 0.05.

## Supplementary information

Suplementary file

## Data Availability

The authors declare the main data supporting the findings of this study are available within this article and its supplementary data.
