# Author Correction: Designing a whole cell bioreporter to show antioxidant activities of agents that work by promotion of the KEAP1–NRF2 signaling pathway

**DOI:** 10.1038/s41598-020-74319-y

**Published:** 2020-10-28

**Authors:** Negar Mozaheb, Ehsan Arefian, Mohammad Ali Amoozegar

**Affiliations:** 1 Extremophiles Lab, Department of Microbiology, School of Biology and Center of Excellence in Phylogeny of Living Organisms, College of Science, University of Tehran, 1417466191 Tehran, Iran; 2 Department of Microbiology, School of Biology, College of Science, University of Tehran, 1417466191 Tehran, Iran

Correction to: *Scientific Reports* 10.1038/s41598-019-39011-w, published online 1 March 2019

The original version of this Article contained a typographical error in the spelling of the author Negar Mozaheb, which was incorrectly given as Negar Mozahheb. This has now been corrected in the PDF and HTML versions of the Article, and in the accompanying Supplementary Information file.

