# Supplementary material for: Designing a whole cell bioreporter to show antioxidant activities of agents that work by promotion of the KEAP1–NRF2 signaling pathway
Source: Sci Rep. 2019 Mar 1;9:3248. doi: 10.1038/s41598-019-39011-w (PMC6397309; doi:10.1038/s41598-019-39011-w)
Supplement: Supplementary file 1 — Suplementary file [file 41598_2019_39011_MOESM1_ESM.docx]

# Designing a whole cell bioreporter to show antioxidant activities of agents that work by promotion of the KEAP1–NRF2 signaling pathway

Negar Mozahheb^1^, Ehsan Arefian^2^*, Mohammad Ali Amoozegar^1^.

1. Extremophiles Lab, Department of Microbiology, School of Biology and Center of Excellence in Phylogeny of Living Organisms, College of Science, University of Tehran, Iran.
2. Department of Microbiology, School of Biology, College of Science, University of Tehran, Iran.

Corresponding authors *

**Supplementary Table S1**. Primer. Primers sequences for NRF2 gene and isolation of promoter-containing region.

| **Reverse primer sequence** | **Forward primer sequence** |  |  |
| --- | --- | --- | --- |
| CATACCGTCTAAATCAACAGG | TGCCCACATTCCCAAATC | **Nrf_2_ primer** | **Real-time Primers** |
| ATCTTCAAACCTCCATGATG | ATGCCTGCCGTGTGAAC | **ß2M primer** |  |
| TCGACTCGAGCTTGAAGTCTTCTCTGGGCAATG | AGCTAAGCTTGGAGTTCGGACGCTTTGAAAC | **-728/+152(insert1)** | **Selected Regions as Response elements** |
| TCGACTCGAGGGAACTGAGGTCGCCTTCG | AGCTAAGCTTCGCAACTCTTACCCTTGACAG | **+75/+460 (insert2)** |  |
| TCGACTCGAGGAGTTGGCCCCTCGTAAC | AGCTAAGCTTCTACACTCGCAACTCTTACCC | **+75/+741(insert3)** |  |


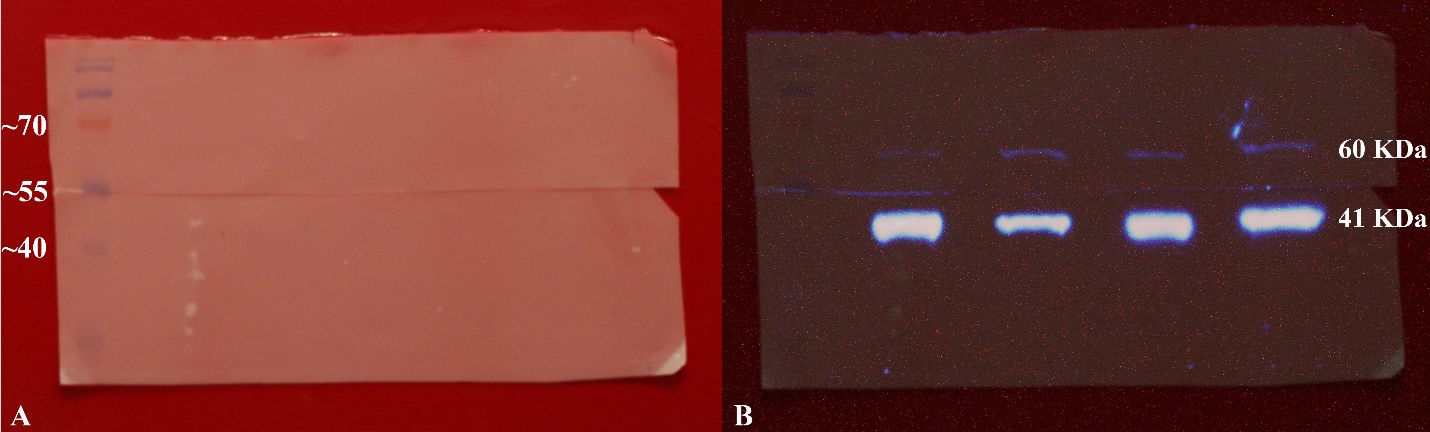


**Supplementary Figure S1.** Supplementary figure for western blot (Fig. 5) with the molecular marker. **A:** Pre-stained Protein Ladder. **B:** Non-cropped picture of blots and protein ladder.
